# Neck dissections: radical to conservative

**DOI:** 10.1186/1477-7819-3-21

**Published:** 2005-04-18

**Authors:** K Harish

**Affiliations:** 1 Professor & Head, Department of Surgical Oncology, M. S. Ramaiah Medical College & Hospital, Bangalore – 560054, India

## Abstract

**Background:**

Neck dissection is an important surgical procedure for the management of metastatic nodal disease in the neck. The gold standard of neck nodal management has been the radical neck dissection. Any modification in the neck dissection is always compared with this standard. Over the last few decades, in order to alleviate the morbidity of radical neck dissection, several modifications and conservative procedures have been advocated. These procedures retain certain lymphatic or non-lymphatic structures and have been shown not to compromise oncological safety.

**Methods:**

A literature search of the Medline was carried out for all articles on neck dissections. The articles were systematically reviewed to analyze and trace the evolution of neck dissection. These were then categorized to address the nomenclature, management of node positive and node negative neck including those who had received chemoradiation.

**Results:**

The present article discusses the neck nodal nomenclature, the radical neck dissection, its modifications and migration to more conservative procedures and possible advances in the near future.

**Conclusion:**

Radical neck dissection is now replaced with modified radical neck dissections in most situations. Attempts are being made to replace modified radical neck dissections with selective neck dissections for early node positivity. Sentinel node biopsy is being studied to address the issue of node negative neck. More conservative surgeries are likely to replace the 'radical' surgeries of bygone era. This process is facilitated by earlier detection of the disease and better understanding of cancer biology.

## Background

Gasparo Aselli, in 1622, first described the lacteal vessels indicating the presence of a lymphatic system. Paolo Mascagni, in 1787, and Sappey, in 1875, published atlases on lymphatics. Most of the later knowledge was derived from autopsy or post-surgical studies on nodes. The standard anatomical depictions of nodes and their drainage were based on Rouviere's descriptions. Surgical removal of nodes has paralleled the anatomical understanding. From these *in vitro *autopsy or post surgical studies on lymph drainage, we have progressed to *in vivo *sentinel node evaluation.

In the early 1800s, complete removal of disease was considered impossible once cancer had spread to 'cervical glands', although Warren in 1847 described an operation for removal of metastatic neck nodes. Butlin advised removal of nodes through a Köcher incision. A systematic operative procedure for removal of cervical lymphatics and nodes, and the first description of radical neck dissection (RND) based on anatomical principles, was described by George Crile Sr., in 1906 [1]. Hayes Martin (the father of modern head and neck cancer surgery) and his associates standardized the technique of RND [2]. Both of these surgeons followed Halsted's principles which were popular at that time. The RND is still considered by some to be the 'gold standard' treatment for neck nodes. However, modifications of the procedure are accepted and are being performed with increasing frequency. Research into breast cancer has contributed a great deal to the understanding of cancer as a systemic disease in contrast to the earlier Halstedian principles that cancer followed an anatomical step-wise progress. Suarez proposed that muscles, vessels and nerves could be preserved without adversely affecting regional control and described 'functional neck dissection' [3]. With increasing knowledge of tumor biology, the next stage in the surgery of metastatic neck disease was the promotion of the functional neck dissection by Bocca [4]. A comparable recurrence rate compared with that of RND was also claimed [5]. Later studies have shown that there is no compromise on oncological effectiveness even though there is deviation from the previous standard *en bloc *resection.

## The regional lymphatics

Regional lymphatic drains from the scalp and skin of head and neck region, the mucosa of the upper aero-digestive tract, salivary glands, and the thyroid gland to specific regional lymph nodal groups. In addition, tumor dissemination via regional lymphatics to these lymph node groups occurs in a predictable and sequential fashion. Hence, specific regional lymph node groups should be appropriately addressed in treatment planning for a given primary tumor site. The anatomy of the nodal involvement and the biology of the disease have to be considered in planning treatment.

**Figure 1:**
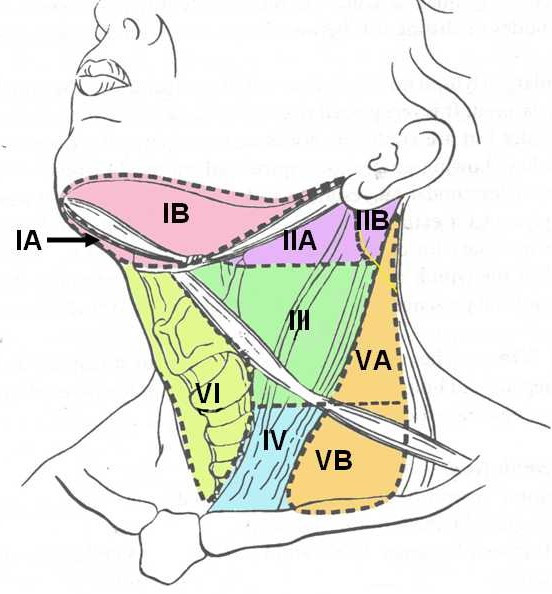
Diagrammatic representation of the neck showing various nodal levels and sublevels

At this stage it is essential that we understand the division of neck nodes into 'nodal groups' described as 'levels' rather than 'anatomical regional groups'. This serves the dual purpose of understanding the natural anatomical spread of disease and the principles of various neck dissections. The nomenclature has been standardized for uniformity in documentation and cross-communication. Retrospective studies have documented the patterns of spread of cancers from various primary sites in the head and neck area to cervical nodes [6]. The terminology for categorizing the lymph node groups was originally described at Memorial Sloan-Kettering Cancer Centre, New York, and is widely accepted. It divides the hemi-neck into five regions. A sixth region was added later [7]. The nodal levels are diagrammatically shown in figure 1 and a brief description of neck nodal levels [8] is described in Table 1. The regional lymph node staging [9] is described in Table 2. Thus, all regional nodes are not at risk of metastases initially from any primary site in the absence of grossly palpable nodes. For example, in oral tumors, if neck is clinically negative, level IV and V nodes are generally not at risk of harboring micro-metastases. A similar situation applies to other organ and node metastases. The first nodal basin varies for each site and is described in the Table 3. Skip metastases in the absence of first echelon nodes being involved are unusual. On the other hand, when clinically palpable nodes are present, comprehensive clearance of all regional nodal groups is warranted (see also discussion under selective dissection for node positive neck). Several well documented studies have confirmed this to be the case [7].

**Table 1:** Cervical nodal levels

**Levels**	**Nodes**	**Boundaries**			
		
		**Superior**	**Inferior**	**Anterior (medial)**	**Posterior (lateral)**
**IA Submental group**	Submental nodes	Symphysis of mandible	Body of hyoid	Anterior belly of contralateral digastric muscle	Anterior belly of ipsilateral digastric muscle
**IB Submandibular group**	Submandibular nodes	Body of mandible	Posterior belly of digastric muscle	Anterior belly of digastric muscle	Stylohyoid muscle
**IIA Upper Jugular group Nodes**	Nodes around upper portions of Internal jugular vein and Accessory Nerve	Skull base	Horizontal plane defined by the inferior border of hyoid bone	Stylohyoid muscle	Vertical plane defined by the spinal accessory nerve
**IIB Upper Jugular group Nodes**		Skull base	Horizontal plane defined by the inferior border of hyoid bone	Vertical plane defined by the spinal accessory nerve	Lateral border of the sternocleidomastoid muscle
**III Mid Jugular group**	Nodes around mid portions of Internal jugular vein	Horizontal plane defined by the inferior border of hyoid bone	Horizontal plane defined by the inferior border of cricoid cartilage	Lateral border of the sternohyoid muscle	Lateral border of the sternocleidomastoid muscle or sensory branches of the cervical plexus
**IV Lower Jugular group**	Nodes around lower third of IJV	Horizontal plane defined by the inferior border of cricoid cartilage	Clavicle	Lateral border of the sternohyoid muscle	Lateral border of the sternocleidomastoid muscle or sensory branches of the cervical plexus
**VA Posterior triangle group**	Nodes around lower part of Accessory nerve and transverse cervical vessels	Apex of the convergence of sternocleidomastoid and trapezius muscles	Horizontal plane defined by the inferior border of cricoid cartilage	Lateral border of the sternocleidomastoid muscle or sensory branches of the cervical plexus	Anterior border of the trapezius muscle
**VB Posterior triangle group**		Horizontal plane defined by the inferior border of cricoid cartilage	Clavicle	Lateral border of the sternocleidomastoid muscle or sensory branches of the cervical plexus	Anterior border of the trapezius muscle
**VI Central or anterior group**	Nodes surrounding midline visceral structures of neck	Hyoid bone	Suprasternal	Common carotid artery	Common carotid artery

**Table 2:** Regional lymph node staging

Nx	Regional lymph nodes cannot be assessed
N0	No regional lymph node metastasis
N1	Metastasis in a single ipsilateral lymph node, 3 cm or less in greatest dimension
N2a	Metastasis in a single ipsilateral lymph node, more than 3 cm but less than 6 cm in greatest dimension
N2b	Metastasis in a multiple ipsilateral lymph nodes, none more than 6 cm in greatest dimension
N2c	Metastasis in bilateral or contralateral lymph nodes, none more than 6 cm in greatest dimension
N3	Metastasis in a lymph node more than 6 cm in greatest dimension (Midline nodes are considered ipsilateral nodes)

(Adapted from UICC / AJCC TNM Classification of Malignant Tumors, 6^th ^edition, 2002)

**Table 3:** Patterns of neck nodal metastasis

**Primary site**	**First echelon nodes**
Oral cavity	Levels I, II, III
Larynx, Pharynx	Levels II, III, IV
Thyroid	Levels IV, VI, superior mediastinal
Parotid	Levels II, III, Pre-auricular, Peri & intra parotid, Upper accessory chain
Submandibular, sublingual glands	Level I, II, III

The risk of nodal metastases is dependent on various factors related to the primary tumor. These include the site, size (large or small), T stage, location of the primary tumor (within an organ such as the vocal cord compared with supraglottis) and histomorphology of the primary tumor. The risk of metastases increases as one progresses from the anterior to posterior part of the upper aero-digestive tract; from lip (10%) progressing along the tongue (25%), gum (30%), floor of mouth (40%), oropharynx (55%) to hypopharynx (65%). Endophytic tumors, poorly differentiated tumors, and tumors with a greater thickness (tongue and floor of mouth) are more likely to have metastases [10].

The goal of treatment of cervical nodal metastases is regional control of disease. Micrometastases and minimal gross metastases may be controlled by radiotherapy but surgery remains the mainstay of treatment as it provides comprehensive clearance of all grossly enlarged nodes and offers accurate histological information regarding the presence of micrometastases. While the indications for comprehensive surgical clearance of regional nodes in patients with clinically metastatic disease are obvious, the indications for elective nodal dissection (END) in patients with N_0 _neck status are more challenging and are discussed separately. The present discussion is aimed at answering why, when, and how to treat neck nodes in patients with primary squamous head and neck cancers?

## Neck dissection nomenclature

The term "neck dissection" refers to a surgical procedure in which the fibro-fatty soft tissue content of the neck is excised to remove the lymph nodes that are contained therein [11]. Over the years, a number of variations and modifications of RND have been described. In addition, different terminologies have been used to represent the same surgery as well as different surgeries. The classification by the American Academy of Head and Neck Surgery, and which is also endorsed by American Society of Head and Neck Surgery, has become accepted widely [7]. The principles are as follows:

1. RND is the standard basic procedure for cervical lymphadenectomy, and all other procedures represent one or more modifications of this procedure.

2. When the modification involves preservation of one or more non-lymphatic structures, the procedure is termed a modified RND (MRND)

3. When the modification involves removal of additional nodal groups or non-lymphatic structures relative to RND, the procedure is termed extended RND.

4. When the modification involves preservation of one or more lymph node groups that are routinely removed in RND, the procedure is termed as selective neck dissection (SND).

The first three procedures are also grouped together under the name of "comprehensive neck dissections". A modified classification is outlined in Table 4. An example of extended neck dissection includes removal of parapharyngeal or buccinator or superior mediastinal nodes. It could also involve removal of non-lymphatic structures such as the hypoglossal or vagus nerves, paraspinal muscles or carotid artery.

**Table 4:** Classification of neck dissections*

Comprehensive neck dissection	Radical Neck Dissection
	Extended Radical Neck Dissection
	Modified Radical Neck Dissection type I
	Modified Radical Neck Dissection type II
	Modified Radical Neck Dissection type III
Selective neck dissection	Supraomohyoid neck dissection
	Jugular (antero-lateral) neck dissection
	Central compartment (anterior) neck dissection
	Posterolateral neck dissection

*The nomenclature of various selective neck dissections are not recommended for usage. They are replaced with SND followed by the nodal levels or sublevels removed. However these named procedures are retained here as this is a transition phase and this nomenclature was being followed not very long ago.

Other terminologies used are "elective" and "therapeutic" neck dissections. If a neck dissection is carried out when there is no evidence of neck disease it is termed an "elective" neck dissection (END). Some authors use the word "prophylactic" instead of "elective" to denote the same procedure. However, the word "prophylactic" is not recommended as it gives a false impression. If the neck dissection is undertaken for metastatic disease in the neck it is called a "therapeutic" neck dissection. An END is usually, but not always, a SND. Hence END and SND are not synonymous terms

## Management of positive neck nodes

Palpable cervical nodes in patients with carcinoma of the upper aero-digestive tract are usually assumed to be due to metastases. Further evaluation with FNAC is needed only in patients where the nodes do not appear to be due to metastatic diseases as assessed clinically. At the other extreme, computerized tomography (CT) scan or magnetic resonance imaging (MRI) or a Doppler study may be required depending on what vital structure appears to be involved by nodal disease, or when there is a doubt about the ability to surgically remove the disease.

The presence of nodal metastases reduces survival by 50% [12,13]. The prognosis of patients with cervical metastases is affected by the number of metastases, level of metastases, tumor burden, presence of extra capsular spread, nodal resectability and previous treatment with surgery or irradiation [12]. With the exception of patients with nasopharyngeal carcinomas, clinically apparent cervical lymph node metastases should be treated with a neck dissection [14].

A classical RND has been regarded as the gold standard for surgical treatment of clinically apparent metastatic cervical lymph node disease. When regional metastases are clinically palpable, comprehensive clearance of all the regional nodes which are at risk is mandatory. The underlying principle is that once metastases have occurred to one node, than there is likely to be sub-clinical metastases in other nodes. However, the operation has a significant morbidity which includes shoulder syndrome (shoulder weakness, deformity, pain, and adhesive capsulitis), anesthesia over the cutaneous distribution of the cervical plexus, and cosmetic deformity due to loss of the sternocleidomastoid muscle. The procedure involves removal of all ipsilateral cervical lymph node groups extending from the body of the mandible superiorly, to the clavicle inferiorly, anteriorly from the lateral border of sternohyoid, hyoid, contralateral anterior belly of digastric muscle belly, to the anterior border of trapezius posteriorly. Nodal groups from level I through to V are included in this dissection. Important extra-nodal structures such as the spinal accessory nerve, internal jugular vein and sternocleidomastoid muscle are sacrificed. Post- auricular, periparotid, suboccipital, perifacial, buccinator, retropharyngeal and paratracheal nodes are not removed in this nodal dissection.

The current indications for classical RND [15] are:

• N3 neck disease, especially in the upper neck

• Bulky metastatic disease near the accessory nerve

• Tumor directly involving the accessory nerve

• Clinically palpable multiple nodes, especially if near the accessory nerve (N2b, N2c)

• Recurrent metastatic tumor after previous irradiation therapy

• Recurrent disease in the neck after previous neck dissection

• Salvage surgery in patients after chemo-irradiation therapy, especially in those who presented with bulky or level II nodal disease

• Involvement of the platysma or skin, requiring sacrifice of a portion of skin in the upper neck

• Clinical or radiological signs of obvious extra-nodal disease e.g. fixity

On the other hand, when appropriate indications exist, a function-preserving comprehensive neck dissection, sparing one or more vital structures, should be considered as long as it does not compromise the performance of a satisfactory clearance of metastatic disease. Preservation of the accessory nerve alone significantly reduces the morbidity of neck dissection. Thus, if the spinal accessory nerve is not involved by metastatic disease, it should be routinely preserved even in patients with clinically palpable metastatic nodes as the accessory nerve is rarely invaded by metastatic disease, and its preservation does not affect local recurrence of disease or overall patient survival. The cervical plexus contributes in part to the motor supply of trapezius through the accessory nerve [16-18]. However, it has also been pointed out that such a contribution may be insignificant and inconsistent [19]. Spinal accessory nerve dysfunction occurs even after selective dissection and is usually attributed to stretching of the nerve due to retraction during clearance of level IIb lymph nodes and/or ischemia [20,21]. Recent anatomical findings have shown that, functionally, the most important descending part of the trapezius muscle is innervated by a fine single branch arising from the spinal accessory nerve in the posterior triangle of the neck. Preservation of this branch may help to prevent more morbidity in patients undergoing modified radical neck dissections [22]. Accessory nerve sparing should not result in less than optimum nodal disease clearance and the nerve should be sacrificed if warranted in order to achieve this. Preservation of the sternocleidomastoid muscle, or internal jugular vein, is not recommended in patients with palpable nodes from an upper aero-digestive tract primary tumor. However, the same can be preserved, in addition to the accessory nerve, in nodal metastases from a well differentiated thyroid carcinoma. In addition, the sternomastoid can be preserved for cosmesis in young individuals and in those with minimal nodal disease. When bilateral neck dissection is undertaken simultaneously, it is important to preserve at least one internal jugular vein, preferably on the less involved side. It has also been realized that neck dissection is inadequate when employed as a sole modality in disease with adverse histological features such as perineural invasion and extracapsular spread. This has led to the usage of combination therapy of surgery with irradiation in selected patients. The common indications for RND, MRND and structures removed or spared are listed in Table 5. The overall results comparing RND and MRND [10,23-31] irrespective of the primary tumor are listed in Table 6. From the above discussion it is apparent that spinal accessory nerve preservation lessens the morbidity to a great extent and preservation of sternocleidomastoid muscle and internal jugular vein is of lesser importance. However, this issue becomes of significance where bilateral neck dissections are required.

**Table 5:** Comprehensive neck dissection*

**Neck dissection**	**Indication**	**Comments**
**RND**	Clinically metastatic neck nodes of upper aero-digestive tract	Sternocleidomastoid, accessory nerve, Internal Jugular vein and submandibular gland removed
**MRND TYPE I**	As above, when accessory nerve away from nodal disease	Accessory nerve spared. Rest as RND
**MRND TYPE II**	Thyroid well differentiated cancer, nodes selectively involving IJV	Sternocleidomastoid, accessory nerve spared. Internal Jugular vein sacrificed. Rest as RND
**MRND TYPE III**	Thyroid well differentiated cancer	Sternocleidomastoid, accessory nerve, Internal Jugular vein spared
**EXTENDED RND**	Extensive involvement of nodes beyond usual levels or involvement of contiguous organs	Additional lymph nodes** or other non lymphatic structures removed

IJV: internal jugular vein; RND: radical neck dissection

* All procedures involve removal of nodes from level I through V

** Nodal areas include retropharyngeal, parapharyngeal, mediastinal or axillary, Structures include cranial nerves, carotid artery, muscles, skin etc.

Note: It is recommended to uniformly use the term modified radical neck dissection (MRND) and not classify as I or II or III but instead name the structures preserved.

**Table 6:** Overall results (irrespective of site of primary tumor)

**Nodal status**	**Neck dissection**	**Neck failure**
**N+**	RND	10% – 22%
	MRND type I	4.8% – 26%
**N1**	RND	8% – 15%
	MRND type I	0 – 16%
**N2**	RND	12% – 26%
	MRND type I	15% – 25%
**N3**	RND	21%

RND: radical neck dissection; MRND: modified radical neck dissection

The outlook for patients with "fixed" nodes is poor [12,32]. The poor prognosis is probably due to an incomplete resection and there being extracapsular spread of disease. The term "fixed node" is vague in the sense it conveys different meaning to different people unless it is known to what structure is the node "fixed". True fixation occurs in 23 to 50% of patients [32]. Tumors fixed to mandible, larynx, sternocleidomastoid, prevertebral muscles and mastoid process can be resected with a margin of normal tissue. Invasion of skin can similarly be addressed by a wide margin of resection with resurfacing then carried out. Adjuvant irradiation can then be employed for microscopic disease. However, the 5-year survivals are poor and are reported to be around 15% [32]. Irradiation followed by salvage surgery did not improve survival and the concept of preoperative irradiation 'to make an inoperable tumor' "operable" is questionable [32]. Invasion of the carotid sheath does not preclude surgery and can be present in 5% of patients [32,33]. Carotid artery invasion is also associated with a poor prognosis. When the external carotid artery alone is involved, it can be sacrificed. When the common or internal carotid is involved, the options are either no treatment, debulking with adjuvant therapy, mixed beam therapy or resection with or without revascularization. Patients should undergo cerebral blood flow studies prior to surgery and elective carotid artery resection can be undertaken. Earlier, carotid resection carried a mortality of 12% and hemiplegia rate of 33% [34]. Recently 22% 2 year disease-free survivals, with acceptable morbidity, have been described [35]. A median disease-specific survival of 12 months, with 24% of patients dying from distant metastases has been reported. The high-risk of complications, loss of quality of life and mortality, must be balanced against the natural history of the disease if it is left untreated [36]. When cervical spine or brachial plexus is involved, sacrifice of these with their attendant morbidities should be weighed against the local control of disease that could be achieved and the benefits that it will offer to patients.

Bilateral neck disease at initial presentation is a bad prognostic sign with a 5-year survival of only 5% then being likely [13]. Survival is not affected by laterality but by nodes larger than 6 cm in size [37]. Therefore, the presence of bilateral mobile nodes should not preclude treatment. At least one internal jugular vein should be preserved or reconstructed with a saphenous graft [38] or polytetrafluoroethelyene graft. Simultaneous ligation of both internal jugular veins can cause venous congestion, edema of the head and neck, raised intracranial pressure and the syndrome of inappropriate secretion of anti-diuretic hormone. Salvage surgery following a previous neck dissection also carries a dismal outlook, with a long-term survival of less than 5% [39].

As an extension of conservation surgery, some authorities advocate SND for metastatic neck nodes [40,41]. These have been only applied in cases without evidence of massive lymphadenopathy, node fixation or gross extra-capsular spread. In addition, when a suspicious node is encountered in the lower limit of the dissection, a formal RND or its modifications can be undertaken. Although the primary site in these cases varies, it has been reportedly performed in low grade buccal or buccoalveolar lesions by an experienced surgeon. SND, when used in combination with postoperative irradiation therapy, has been shown to achieve comparable results to those reported for RND [42]. However, as a routine, a SND is not recommended for patients whose neck nodes are positive for tumor.

The role of neck dissection after definitive chemoradiotherapy for squamous cell head and neck cancer is not clearly defined [43]. After chemoradiotherapy (5 Fluorouracil and cisplatin), clinical parameters do not identify those patients with residual neck node disease or those at risk for regional failure, suggesting that neck dissection should be considered for all patients with N2-N3 disease [43]. A retrospective non- randomized study showed that the 5-year survival rate was significantly higher for those who had postoperative radiotherapy (38.9%) compared with patients who had pre-operative radiotherapy (9.1%) and those having surgery alone (0%). They were unable to evaluate the role of definitive chemotherapy and/or radiotherapy and salvage surgery as the results were inconsistent and the available data was limited [44].

Surgical resection is an essential component of aggressive chemoradiation protocols to ensure tumor control at the primary site and also in the neck. Neck dissections in patients with initial node positive disease reveal residual nodal metastases in 22% [45]. A German multicenter randomized trial showed that accelerated chemoradiation (Mitomycin and 5 Fluorouracil) to 70.6 Gy was more effective than accelerated radiation to 77.6 Gy alone. Selective lymph node dissection of residual neck masses after completion of hyperfractionated accelerated radio-(chemo-)therapy is likely to contribute to loco-regional tumor control in patients with advanced head and neck cancers [46]. On the contrary, another study did not recommend neck dissection for patients who respond completely after chemoradiation but did recommend it for patients with either no or partial radiographic responses [47]. Another study showed improved neck control with neck dissection for patients with clinically residual disease or N3 neck cancer but no significant impact on the outcome of patients with N2 stage disease who were rendered clinically disease-free with intensive concurrent chemoradiation [48]. There is also a suggestion that neck dissection followed by chemoradiotherapy has a higher, and statistically significant, disease-specific survival rate compared to chemoradiation alone [49]. Consensus regarding the use of neck dissections for complete responders and incomplete responders has yet to be achieved and the data are controversial. A possible benefit from neck dissection after a complete response of the primary tumor after combined chemoradiation or definitive external radiation for advanced squamous cell carcinoma of the head and neck may only be anticipated in patients with persisting sub-clinical neck disease who have no other sites of disease. Some clinicians have even argued that the salvage rate for clinically detectable residual neck disease does not justify neck dissection being performed. Randomized trials addressing these questions, and a trial addressing the accuracy of new imaging modalities such as post chemotherapy and post radiation positron emission tomography scanning, across multiple institutions would be appropriate [50,51].

Pathological evaluation of the neck dissection specimens is important. A RND or MRND specimen should comprise of at least 10 nodes on pathological examination. Apart from the number of involved nodes it is important to identify adverse prognostic factors resulting in a predisposition to local recurrence and a reduced survival. Perineural invasion, extracapsular spread and vascular invasion indicate a bad prognosis [52,53]. Desmoplastic stromal reaction was associated with a seven fold increase in the risk of recurrent disease. Tumor has been identified up to 12 cm along the perineural space in patients with perineural invasion, and should be considered as a positive tumor margin [2]. Patients with poor prognostic factors listed above should receive adjuvant therapy such as irradiation, with or without chemotherapy, as required.

### Management of patients with N0 neck status

As discussed earlier, comprehensive neck dissection is the standard treatment for obvious nodal disease in the neck. With emphasis on function preservation and cosmesis in addition to achieving adequate local disease control, conservative neck dissections have gained popularity. Growing historical evidence suggests that modified and selective neck dissections offer disease control comparable to radical neck dissection but with less morbidity [6,40,54,55].

#### The problem

It is well known that 20% to 40% of patients with head and neck cancer who have no palpable disease in their necks will harbor occult disease in their necks. It is, therefore, therapeutically tempting to treat the neck in these patients on the basis that this will avoid subsequent treatment, which may be not as effective. It means that 60%-80% of patients are "over-treated". The pros and cons of this [56-61] are addressed in Table 7.

**Table 7:** Pros and cons for elective neck dissection (END)

**For END**	**Against END**
Neck dissection has low morbidity & mortality	END results in a large number of unnecessary surgical procedures and is associated with inevitable morbidity
Cure rate for neck dissection is decreased if gland enlargement occurs or multiple nodes appear	Cure rates are no lower if the surgeon waits for the neck to convert from N0 to N1
It is impossible to provide follow-up necessary to detect the earlier conversion of a neck from N0 to N1	Careful clinical follow-up will allow detection of the earliest conversion from N0 to N1
Allowing the neck metastases to develop increases the incidence of distant metastasis	END removes the barrier to the spread of disease and also has a detrimental immunological effect
If neck has been entered to remove the primary it is better to perform an in-continuity resection	Radiation is as effective as neck dissection in N0 neck
High incidence of occult metastatic disease	

#### Evaluation of patients with N0 neck status

Cervical nodal staging is a major challenge. Clinical examination is influenced by the skill of the examiner, the patient's body habitus and whether the patient has had previous surgical or irradiation therapy. As a result of these factors, the false negative rate in clinical assessment ranges from 20%-51% [62]. Imaging techniques such as CT scanning, MRI and ultrasound have been used for evaluation. The sensitivity of CT scanning is 81% [63]. Size of nodes and central lucency of nodes have been used as criteria for identifying nodes containing metastatic tumor but there is a low specificity [64]. Routine scanning of the neck in a patient with a head and neck primary tumor is not justifiable at present. With ultrasound and ultrasound guided aspiration, the accuracy has improved to 89% but it is highly operator- dependent [65]. No pre-treatment study can accurately assess or replace the requirement to stage the neck histopathologically [63]. Hence the goal of identifying 'sub-clinical disease' without surgical intervention remains elusive.

#### Surgical management

A standard RND has no role in the management of patients with N0 neck status. A SND, or rarely a MRND, would be required. A recent study has shown comparable recurrence rates for RND compared with SND with there being no statistically significant difference [66]. It has to be emphasized that an END procedure must be "individualized". The decision regarding the type of neck dissection depends on the site of primary tumor, other tumor factors (e.g. location, depth, size, differentiation, vascular or perineural invasion), the patient and the surgeon undertaking the procedure. Tumors of the tongue, floor of mouth, nasopharynx, oropharynx, hypopharynx and supraglotic larynx have a high incidence of nodal metastases. In contrast, tumors of the buccal mucosa, lip, paranasal sinuses and glottic larynx metastasize less frequently. For a patient who stays some distance from the surgical centre and who is not expected to come for regular follow-up (and for unreliable patients) an END would be a better option. Documentation of differences in regional control rates or survival between patients undergoing END for N0 disease or patients undergoing therapeutic neck dissection for N1 disease remains controversial. Some authors claim no difference [10] while other studies have shown that END significantly improved regional control of the disease [67,68]. Unfortunately, many patients do not present later with just N1 disease but with disease greater than that of N1 staging [10]. Hence END must always be considered based on the primary tumor characteristics as prognosis depends on the extent of metastatic neck nodal disease. An END accurately stages the disease pathologically, detects and treats micro-metastases, thus controlling regional disease and helping in the planning of adjuvant therapy.

When level IV nodes are removed along with supraomohyoid neck dissection (SOND), as in primary tongue, terminologies like extended SOND have been used. This adds to the complexity of the classification. Further, the classification of types of SND does not have nomenclature options if the surgeon preserves certain nodal sublevels. Hence, it is recommended to use the nodal levels or sublevels which are dissected instead of the named SND [8].

#### Radiation in patients with N0 neck status

External radiation in doses of 40 to 50 Gy will control occult metastases in 90 to 95% of cases [69]. Although prospective evidence is lacking, retrospective data suggest that for most sites and for early lesions, elective nodal irradiation (ENI) and END offers equivalent local control [54,70]. Proponents of ENI assert that the morbidity is low with limited soft tissue changes and does not have systemic ramifications. However, considerable acute adverse effects such as mucositis and xerostomia, together with late effects like endarteritis, radionecrosis etc .can occur. Systemic effects include suppression of humoral and cell mediated immunity.

The issue of the use of elective surgery versus elective radiation ends not at which treatment modality is more beneficial, but which one is less harmful. The patient's age, general health, family support, reliability and patient's own wishes are important. It is impossible to compare elective neck dissection and elective nodal irradiation efficacy because the status of neck disease is unknown when elective irradiation is used. The accurate histological information on micrometastases in neck nodes in patients with clinically negative neck nodes is probably one of the prime factors that tilts the argument towards nodal dissection, apart from the lesser associated morbidity. If the treatment planning for the primary tumor involves surgical excision through a neck approach, END is opted for when indicated. If the primary tumor is being treated with irradiation, an elective irradiation of nodal area should be planned.

Most authors recommend that END be performed when the expected incidence of microscopic or subclinical disease exceeds 20%. The National Cancer Comprehensive Network's adopted practice guidelines recommend END for cancers of the oral cavity, oropharynx, hypopharynx and supraglottic larynx when the nodal status is N0.

Tumors of the nasopharynx are primarily treated with radiotherapy, which includes nodal management. Surgery plays a limited role with this being restricted to salvage of residual neck disease following failure of primary radiotherapy.

For oral tumors, the high incidence of occult nodal metastases, and notoriously poor salvage rates, make expectant treatment ill-advised in most cases [54,55,71]. The lower alveolar ridge carcinomas have a low potential for neck metastases [72]. With the exception of this, treatment of sub-clinical neck disease is indicated for all oral cavity lesions. It is also important to note that carcinomas of the oral cavity rarely spread to lower jugular or posterior cervical nodes in the absence of involvement of level I-III. An elective SND (levels I – III) provides staging information and may be the only therapy necessary for occult disease. It has minimal morbidity and it reduces the risk of occult disease evolving into clinically evident metastases. In addition, the undesirable effects of radiotherapy are avoided. A tongue tumor with a depth of invasion of more than 4 mm is likely to produce occult nodal metastases [73]. As a sub-site of oral tumors, the tongue is notorious for nodal metastases. For a well differentiated T1 tumor, with less than 4 mm of muscle invasion and a clinical N0 status in the neck, a model predicts a 14% chance of nodal metastases. Sub-digastric and mid jugular are the most frequently involved nodal groups in primary oral cavity tumors [65]. Evidence also indicates that level IV nodes may also be at risk in a tongue primary cancer [74]. An ipsilateral SND (levels I – IV) is indicated for tongue primaries. Both sides of the neck have to be addressed in midline, or near the midline, tongue primaries.

In tumors of the oropharynx, the risk of occult nodal metastases is 30%-35%. There is a high incidence of level II, III and IV involvement [55,74]. In addition, there is an increased risk of bilateral occult nodal disease in tumors near the midline and treatment should include both sides of the neck. Tumors of the hypopharynx are known for extensive sub-mucosal spread. The retropharyngeal and paratracheal nodes are also at risk of harboring metastases. Occult nodal metastases are present in approximately 30%-55% of patients with primary hypopharyngeal tumors and occur frequently in the contralateral side of the neck [55,75]. As stated earlier, the modality used in the treatment of the primary is an important consideration whilst planning treatment of the neck. Larger tumors (T3 or greater) usually require combined therapy. In cases where surgery is chosen to treat an advanced primary cancer, a bilateral neck dissection aiming at removal of all the nodes at risk is performed. If combined therapy is planned, a unilateral neck dissection is undertaken and irradiation may be used to treat the un-dissected neck. The choice of treatment is dependent on various factors as discussed earlier.

In tumors of the supraglottic larynx, the location of the tumor has a prognostic significance. Marginal lesions like those on aryepiglottic fold and suprahyoid margin of the epiglottis behave more aggressively with an increased risk of nodal metastases. There is also a high risk of contralateral nodal metastasis for lateral (aryepiglottic fold) as well as midline (epiglottic) lesions. This makes bilateral neck dissections a necessity when electively performed [76]. Carcinoma of the supraglottic larynx has a high incidence of occult metastases in the range of around 30% [55]. Based on current evidence, if surgery is planned for primary therapy, SND of levels II through IV is acceptable [77]. Dissections need to be bilateral to prevent contralateral neck failure and recurrence of disease. As stated earlier, if irradiation is planned, the contralateral side of the neck may be irradiated.

In contrast, glottic cancers remain localized due to sparse lymphatics. T1 and T2 carcinomas have less than 7% nodal metastases. If such patients are available for regular follow-up, these patients may be closely observed. But, recurrent cancers have a significantly higher occult metastases rate reaching 20%-22%. An END incorporating levels II through IV may be planned [55,77]. T3 and T4 glottic carcinomas have occult nodal metastasis in the range of 20%-40%. In addition, salvage rates for recurrences are dismal. Hence, T3 and T4 glottic carcinomas should receive ipsilateral SND of levels II through IV and level VI.

The reported incidence of subglottic tumors is low. These have a high incidence of extralaryngeal spread due to the proximity of the cricothryoid membrane and the rich post-cricoid lymphatics. They drain to pre- and para-tracheal, pre-laryngeal and superior mediastinal nodes. The sparse data makes conclusive recommendations difficult. However, a wide field laryngectomy, bilateral para-tracheal dissection, pre-laryngeal nodal dissection with an ipsilateral hemithyroidectomy should be planned with postoperative radiation to stoma, mediastinum and both sides of neck. An organ based general plan of SND is outlined in Table 8.

**Table 8:** Selective neck dissection (N0 neck)

**Organ**	**Nodal clearance**
Oral cavity	I, II, III
Tongue	I, II, III, !V
Hypopharynx, larynx, oropharynx	II, III, IV
Some laryngeal and hypopharyngeal lesions where IIB is not removed	IIA, III, IV
Laryngeal, hypopharyngeal extending below glottis	II, III, IV, VI
Thyroid, hypopharynx, cervical trachea, cervical esophagus, sub-glottic larynx	VI,
Cutaneous carcinoma of posterior scalp and upper neck	II – V, Post auricular, Suboccipital
Cutaneous malignancy from pre-auricular, anterior scalp and temporal region	II, III, VA, parotid, facial, external jugular nodes
Cutaneous malignancy of anterior or lateral face	I, II, III

A SND should include at least 6 nodes on pathological examination. If nodes appear involved near the lower end of the dissection, the SND should be converted to a formal MRND. Alternatively, if facilities are available, frozen section can be employed.

SND is traditionally looked upon as a diagnostic or staging procedure. Increasing evidence indicates that the procedure could definitely be therapeutic, at least in N0 and early N+ disease, with results comparable to RND [40]. The therapeutic efficacy of SND is not diminished in the presence of occult nodal disease. However, other evidence indicates that recurrence rates are higher if post-operative irradiation is not used when one or more nodes are pathologically positive after SND [78]. SND followed by irradiation if nodal metastases are detected by pathology later is one area under investigation.

#### Trends in management of patients with N0 neck status

*Sentinel node biopsy (SNB) *This new concept is an *in vivo *assessment of tumoral spread to lymph nodes and could be useful in patients with N0 neck status where the problem is whether to treat, or wait and watch. The procedure is similar to that followed for SNB in patients with breast cancer or melanoma, the drawbacks are also similar. The SNB is performed after radiocolloid and blue dye injection (either alone or in combination). Preoperative lymphoscintigraphy and peri-operative use of a gamma probe identifies radioactive sentinel nodes and visualization of blue stained lymphatics and identify the blue sentinel node(s). No further nodal dissection is done if the SNB is negative, whilst therapeutic neck dissection is performed when the SNB is found to harbor malignancy. Various studies have illustrated the feasibility of the procedure in patients with head and neck cancers [79,80]. The sensitivity of the procedure is reported to be 94%. The argument for undertaking such a procedure is that it does not result in an excessive form of treatment whilst at the same time it does not neglect the oncological safety aspects.

A suggestion has been made to divide the occult metastases seen on microscopy in these nodes into isolated tumoral cells, micrometastases and macrometastases. Preliminary results of one trial shows that SNB is of value in patients with T1 and T2 tumors of the oral cavity and oropharynx [81]. Although the procedure holds promise for the future, more randomized studies and long-term follow-up are needed before this procedure is accepted as the standard of care [81-84]. Unresolved issues include identification of patient and tumor characteristics appropriate for this methodology, for example, stage, site or subsite [85]. Another important issue which has to be addressed is whether immunohistochemical or molecular studies are required on SNB and the clinical implication of such micrometastases.

### Endoscopic neck dissections

With the advent of minimally invasive surgery, and after its success in certain abdominal conditions, the same has been tried in other areas of cancer management. Early experiments on animals and cadavers have been fairly successful [86]. Although the feasibility of the procedure has been shown, it's oncological safety and useful in practice are other issues that need to be addressed. The questions to be answered include whether such a procedure benefits the patient to the extent other endoscopic procedures have [87]. It remains to be seen if the procedure gains widespread acceptance in clinical practice.

#### Positron emission tomography (PET) and single photon emission computed tomography (SPECT)

PET may be able to detect nodal metastases in nodes that are judged to be negative by size criteria. SPECT imaging with deoxy-18F-fluoro-D-glucose or thallium is able to detect nodal disease. SPECT using Tc-99 m labeled monoclonal antibodies can visualize most lymph node metastases. However, the accuracy is superior when combined with CT scanning [87]. PET is at present prohibitively expensive but in future it may have a role in detecting occult nodal disease. Further studies are required to correlate the scan results with histological results of node examinations.

## Conclusion

Neck dissection is the standard therapy for patients with metastatic neck nodes. RND has now been replaced with MRND for selected cases of patients with node positive neck disease. The improvements in understanding the biology of cancer and techniques in surgery have led to this change with its associated reduction in morbidity. Imaging studies to predict histological nodal metastases have so far not been successful. Evaluation is underway to replace MRND with SND for early nodal disease. SNB may replace SND for patients with N0 disease in future but certain issues still need to be addressed. Finally, whether endoscopic neck dissections will replace the existing procedures remains to be evaluated.

## List of abbreviations

**RND: **Radical Neck Dissection

**MRND: **Modified Radical Neck Dissection

**END: **Elective Nodal Dissection

**SND: **Selective Neck Dissection

**SNB: **Sentinel Node Biopsy

**ENI: **Elective Nodal Irradiation

**SOND: **Supra Omohyoid Neck Dissection

**PET: **Positron Emission Tomography

**SPECT: **Single Photon Emission Computerized Tomography

## Competing interests

The author(s) declare that they have no competing interests.

## Authors' contributions

**KH **Conceptualized organized the textual material after the literature search and has written the manuscript.
